# Elevated preoperative neutrophil‐to‐lymphocyte ratio predicts early adverse outcomes in uncomplicated type B aortic dissection undergoing TEVAR

**DOI:** 10.1186/s12872-021-01904-y

**Published:** 2021-02-16

**Authors:** Hongqiao Zhu, Lei Zhang, Taiping Liang, Yiming Li, Jian Zhou, Zaiping Jing

**Affiliations:** Department of Vascular Surgery, Changhai Hospital, Navy Medical University, Shanghai, 200433 China

**Keywords:** Neutrophil‐to‐lymphocyte ratio, Thoracic endovascular aortic repair, Uncomplicated type B aortic dissection, Systemic inflammatory response, Inflammatory markers

## Abstract

**Background:**

Thoracic aortic endovascular repair (TEVAR) of uncomplicated type B aortic dissection (uTBAD) has favorable long-term outcomes but higher early adverse events compared with the optimal medical treatment. Recently, clinical evidence concerning vascular surgery indicates that elevated preoperative systemic inflammatory response predicts adverse clinical events. The aim of our study was to evaluate the relationship between preoperative neutrophil-to-lymphocyte ratio (NLR) and early outcomes of uTBAD patients undergoing TEVAR.

**Results:**

216 patients diagnosed with uTBAD were included in this retrospective study between January 2015 and December 2018. The median (IQR) follow-up period was 21 (15–33) months. An early adverse event was defined as occurring within 2 years after the procedure. Median patient age was 60 (IQR, 48–68) years and 78.7 % were male. Early adverse events occurred in 24 patients (11.1 %). In the multivariable analysis, preoperative NLR (HR per SD, 1.98; 95 % CI, 1.14–3.44; *P* = 0.015) was associated with 2-year adverse events.

**Conclusions:**

NLR is an independent predictive factor of early adverse events in uTBAD patients undergoing TEVAR.

## Background

Acute type B aortic dissection (TBAD), one kind of acute aortic syndromes, featured with sudden onset, rapid progression, and high mortality [1, 2]. Despite its name, “uncomplicated” TBAD (uTBAD) is still a problematic disorder that is associated with high long-term mortality when treated medically alone [3]. With the advantages in exclusion of primary entry tear and remodeling of affected aorta, thoracic endovascular aortic repair (TEVAR) was attempted in the uTBAD combined with optimal medical treatment [4]. Clinical researches based on retrospective cohort suggest that TEVAR is beneficial in long-term outcomes for uTBAD [3, 5].

However, there are still debates about the endovascular repair of uTBAD because of disappointing early outcomes compared with optimal medical treatment [6]. Recently, there has been a significant body of work which has investigated the use of systemic inflammatory marker neutrophil-to-lymphocyte ratio (NLR) to both prognosticate patients and to guide treatment [7–9]. In a cross-section study containing 1021 patients receiving major vascular surgery, elevated preoperative NLR (> 5) was an independent risk factor of 2-year mortality [10]. It would therefore be of great interest to clearly define the association with baseline NLR and early clinical outcomes in uTBAD patients undergoing TEVAR.

The study aimed to determine whether elevated baseline NLR is correlated to early poor outcomes of uTBAD patients after TEVAR.

## Methods

### Patient selection

A total of 216 consecutive patients at the Department of Vascular and Endovascular Institution (Changhai Hospital, Shanghai, China) who underwent TEVAR of their primary uTBAD between January 2015 and December 2018 were included in the current study.

Overall exclusion criteria were: patients who were < 18 years old; had aortic dissection secondary to iatrogenic injury, trauma, intramural hematoma or penetrating aortic ulcer; had genetic disease such as Marfan syndrome or Ehlers-Danlos syndrome [11].

Additionally, the following susceptible factors and clinical conditions that may affect baseline systemic inflammatory status or postoperative status were excluded: (1) Patients with proximal landing zone 1 demanding for a hybrid or multi-fenestration technique; (2) Any peripheral artery disease or coronary artery disease being treated with dual anti-platelet therapy; (3) Any malignant disease or end-stage disease like uremia; (4) Any chronic infectious disease being treated with antibiotics; (5) Any autoimmune disease being treated with glucocorticoid [12].

According to the criteria above, 33 patients were excluded, which composed of 5 cases diagnosed with Marfan Syndrome, 8 cases diagnosed with cancer, 12 cases treated with hybrid technique, 1 case with AMI treated with dual anti-platelet therapy, 3 cases treated with dialysis and 4 cases suspected with signs of infection.

Computed tomography angiography (CTA) examinations were performed before the operation to evaluate characteristics of aortic dissection. The follow-up period ended on February 1, 2020.

### Medical management

Medical treatment aimed to relieve the stress of aortic adventitia and reduce the volume of false lumen [6]. Aggressive antihypertensive therapy by oral or intravenous route was used to keep the systolic blood pressure < 120 mmHg and the heart rate < 70 beat/min [6, 11]. Adequate analgesia was administered when patients still felt pain after antihypertensive therapy.

### TEVAR procedure

All patients received general anesthesia in order to administrate blood pressure and heart rate during the procedure. All procedures were performed with patients using total endovascular techniques under fluoroscopic guidance. The proximal landing zone was considered to be at least > 2 cm [13] If the landing zone was inadequate, reconstruction of the left subclavian artery (LSCA) was performed to preserve the blood of the brain and reduce the complications [14].

### Follow‐up

Patients were advised to check up annually in our hospital. Health conditions of patients who did not return were contacted via telephone.

### Baseline blood count

Samples of 216 patients’ complete blood count with differential 24 hours before the TEVAR procedure were accessed from the electronic medical record in Changhai Hospital.

### Statistics analysis

Continuous variables were expressed as the mean ± standard deviation or median (with interquartile range, IQR); categorical variables were expressed as frequencies and percentages. A time-dependent receiver operating characteristic (ROC) curve was utilized to identify a cut-off value NLR associated with 2-year adverse events. Study cohort was then divided into two groups by cut-off value of NLR 4.8. Patients were then grouped into ‘low NLR’ (< 4.8) and ‘high NLR’ (≥ 4.8) groups to identify the differences of clinical characteristics and outcomes. Difference between the two groups were compared by Student’s t-test or Mann-Whitney U test for Continuous variables and a chi-square test for categorical variables. To analyze the relationship between variates and outcomes, univariate and multivariate Cox proportional hazards regression models was used. Variables found to be susceptive (P < 0.2) in univariate analysis were entered into a Cox regression multivariate model using a backwards conditional method. A P value < 0.05 was considered statistically significant and all estimates were two-tailed. All analyses were performed with R (http://www.R-project.org) and Empower Stats software (www.empowerstats.com, X&Y Solutions, Inc., Boston, MA, USA).

### Definition of terms and outcomes

Phase of TBAD was according to the VIRTUE Registry and ESC guideline [4, 11]. “Uncomplicated” is a relatively stable status administrated by optimal medicine, on which the patients are expected to survive without severe comorbidities [11, 15]. The definition of procedure success was to exclude the primary entry tear in the premise that there was no type I or III endoleak at the end of the operation. Post-implantation syndrome (PIS) was defined as fever > 38 °C, WBC > 12.0/nl and CRP > 10 mg/dl within 3 days after TEVAR despite negative blood culture results [16]. The definition of an early adverse event was occurrence within 2 years after the procedure. The primary endpoint was early adverse events, which included all-cause mortality, type I/II endoleak, retrograde aortic dissection (RTAD), stent graft-induced new entry (SINE), paraplegia, major stroke, aortic rupture.

## Results

### Baseline characteristics

The median follow-up time was 21 months (interquartile range, IQR 15–33). The baseline characteristics of this cohort are documented in Table 1. Briefly, 50 % of patients were older than 60, 21.3 % were females. Additional characteristics of the patients included smoking in 27.8 %, alcohol use in 18.1 %, hypertension in 66.2 %, diabetes in 4.6 %, chronic obstructive pulmonary disease (COPD) in 3.7 %, renal insufficiency in 5.1 %, stroke in 4.6 %, and coronary artery disease (CAD) in 6.5 % (Table 1). For the inflammatory status, 48.1 % had high NLR and 51.9 % had low NLR (Table 1). Significant differences was shown between the high and low NLR group in median age (year) (54 vs. 62; *P* = 0.018), portion of partial thrombosis (%) (30.8 % vs. 16.1 %; *P* = 0.02), median systolic blood pressure on admission (mmHg) (148 vs. 140; *P* = 0.007), median serum creatinine before procedure (µmol/l) (81 vs. 76; *P* = 0.036), median platelet count before procedure (×10^9^/l) (170 vs. 221; *P* < 0.001) (Table 1).

**Table 1:** The baseline preoperative characteristics of uTBAD patients receiving TEVAR grouped by NLR value < 4.8 and ≥ 4.8

Variable	Overall	Low NLR (< 4.8)	High NLR (≥ 4.8)	Standardized difference	*P *value
N (%)	216	112 (51.9 %)	104 (48.1 %)		
Age (year)	60 (48–68)	62 (50–70)	54 (46–66)	0.32	0.018
Age group (year)				0.27	0.146
< 40	20 (9.3 %)	10 (8.9 %)	10 (9.6 %)		
40–60	88 (40.7 %)	39 (34.8 %)	49 (47.1 %)		
≥ 60	108 (50.0 %)	63 (56.3 %)	45 (43.3 %)		
Male	170 (78.7 %)	83 (74.1 %)	87 (83.7 %)	0.24	0.087
BMI	24.9 (3.9)	24.9 (4.0)	25.0 (3.8)	0.04	0.764
Smoking	60 (27.8 %)	30 (26.8 %)	30 (28.9 %)	0.05	0.736
Drinking	39 (18.1 %)	20 (17.9 %)	19 (18.3 %)	0.01	0.937
Hypertension	143 (66.2 %)	73 (65.2 %)	70 (67.3 %)	0.05	0.741
Diabetes	10 (4.6 %)	5 (4.5 %)	5 (4.8 %)	0.02	0.904
COPD	8 (3.7 %)	3 (2.7 %)	5 (4.8 %)	0.11	0.408
Renal insufficiency	11 (5.1 %)	8 (7.1 %)	3 (2.9 %)	0.2	0.155
Coronary artery diseases	14 (6.5 %)	9 (8.0 %)	5 (4.8 %)	0.13	0.336
Stroke	10 (4.6 %)	4 (3.6 %)	6 (5.8 %)	0.10	0.442
Dissection morphology				0.04	0.747
Confined in thoracic aorta	56 (25.9 %)	28 (25.0 %)	28 (26.9 %)		
Extended to abdominal aorta	160 (74.1 %)	84 (75.0 %)	76 (73.1 %)		
False lumen patency				0.38	0.021
Patent false lumen	156 (72.2 %)	90 (80.4 %)	66 (63.5 %)		
Partial thrombosis	50 (23.2 %)	18 (16.1 %)	32 (30.8 %)		
Complete thrombosis	10 (4.6 %)	4 (3.6 %)	6 (5.8 %)		
Symptoms on admission				0.09	0.81
Chest/back pain	156 (72.2 %)	79 (70.6 %)	77 (74.0 %)		
Abdominal pain	52 (24.1 %)	29 (25.9 %)	23 (22.1 %)		
Other symptoms	8 (3.7 %)	4 (3.6 %)	4 (3.9 %)		
Location of the primary entry tear				0.16	0.231
> 2 cm from the LSCA	169 (78.2 %)	84 (75.0 %)	85 (81.7 %)		
≤ 2 cm from the LSCA	47 (21.8 %)	28 (25.0 %)	19 (18.3 %)		
Maximum diameter of thoracic aorta (mm)	40.7 (35.2–44.3)	39.7 (35.0–44.1)	40.8 (36.5–44.3)	0.09	0.517
Maximum diameter of abdominal aorta (mm)	28.7 (26.6–32.9)	28.5 (26.5–32.4)	29.4 (26.6–32.9)	0.16	0.248
SBP on admission (mmHg)	143 (132–154)	140 (130–150)	148 (136–157)	0.37	0.007
DBP on admission (mmHg)	80 (75–89)	82 (75–89)	80 (75–88)	0.04	0.77
Temperature preoperatively (℃)	36.6 (36.4–36.8)	36.6 (36.4–36.9)	36.6 (36.4–36.8)	0.24	0.077
Creatinine preoperatively (µmol/l)	79 (64–91)	76 (59–86)	81 (65–95)	0.29	0.036
Platelet count preoperatively (×10^9^/l)	200 (152–272)	221 (169–302)	170 (149–225)	0.42	0.003

Categorical variables are reported as frequency and percentage; continuous variables are reported as median (25th–75th percentile)

*uTBAD* uncomplicated type B aortic dissection,* TEVAR* thoracic endovascular aortic repair,* NLR* neutrophil-to-lymphocyte ratio,* BMI* body mass index, *COPD* chronic obstructive pulmonary disease, *LSCA* left subclavian artery, *SBP* systolic blood pressure, *DBP* diastolic blood pressure

### Medical managements

Details of medical treatment was shown in Table 2. There was no difference between the two groups except calcium-channel blockers (high NLR group vs. low NLR group, 84.6 % vs. 69.6 %, *P* = 0.009).

**Table 2:** The details of medical managements grouped by NLR value < 4.8 and ≥ 4.8

Variable	Overall	Low NLR (< 4.8)	High NLR (≥ 4.8)	Standardized difference	*P* value
Alpha-blockers	166 (76.9 %)	87 (77.7 %)	79 (76.0 %)	0.04	0.765
Beta-blockers	140 (64.8 %)	69 (61.6 %)	71 (68.3 %)	0.14	0.306
ARBs	78 (36.1 %)	43 (38.4 %)	35 (33.7 %)	0.1	0.469
ACEIs	32 (14.8 %)	15 (13.4 %)	17 (16.4 %)	0.08	0.542
CCBs	166 (76.9 %)	78 (69.6 %)	88 (84.6 %)	0.36	0.009
Statins	86 (39.8 %)	44 (39.3 %)	42 (40.4 %)	0.02	0.869

Values are n (%)

*ARBs* angiotensin II receptor blockers, *ACEIs* angiotensin-converting enzyme inhibitors, *CCBs* calcium-channel blockers, *NLR* neutrophil-to-lymphocyte ratio

### TEVAR procedure

27 adjunctive stents were implanted to reconstruct the LSCA. Details of the procedure were shown in Table 3.

**Table 3:** The details of TEVAR procedure grouped by NLR value < 4.8 and ≥ 4.8

Variable	Overall	Low NLR (< 4.8)	High NLR (≥ 4.8)	Standardized difference	*P* value
Length of hospital stay (days)	10 (8–14)	10 (7–14)	10 (9–15)	0.24	0.076
Timing of procedure				0.05	0.713
Acute phase	126 (58.3 %)	64 (57.1 %)	62 (59.6 %)		
Subacute phase	90 (41.7 %)	48 (42.9 %)	42 (40.4 %)		
ASA Classification				0.24	0.205
I	68 (31.5 %)	33 (29.4 %)	35 (33.7 %)		
II	140 (64.8 %)	77 (68.8 %)	63 (60.6 %)		
III	8 (3.7 %)	2 (1.8 %)	6 (5.7 %)		
Length of procedure (minutes)	95(75–110)	95(80–122)	85(72–105)	0.31	0.025
Types of stentgraft					
Cook Zenith	93 (43.1 %)	48 (42.9 %)	45 (43.3 %)	0.4	0.044
Gore TAG	54 (25.0 %)	33 (29.4 %)	21 (20.2 %)		
Medtronic Valiant	55 (25.5 %)	21 (18.8 %)	34 (32.7 %)		
Microport Hercules	14 (6.4 %)	10 (8.9 %)	4 (3.9 %)		
Adjunctive stents	27 (12.5 %)	15 (13.4 %)	12 (11.5 %)	0.06	0.681

Values are median (25th–75th percentile) or n (%)

*TEVAR* thoracic endovascular aortic repair, *NLR* neutrophil-to-lymphocyte ratio, *ASA* American Society of Anesthesiologists

### Early adverse events

There were no in-hospital adverse events in either group. PIS occurred in 8 (3.7 %) of 216 patients with no difference between the two groups (4.8 % for high NLR group and 2.7 % for low NLR group, *P* = 0.41). None of the patients above suffered from the adverse events within 2-year follow up.

**Fig. 1:**
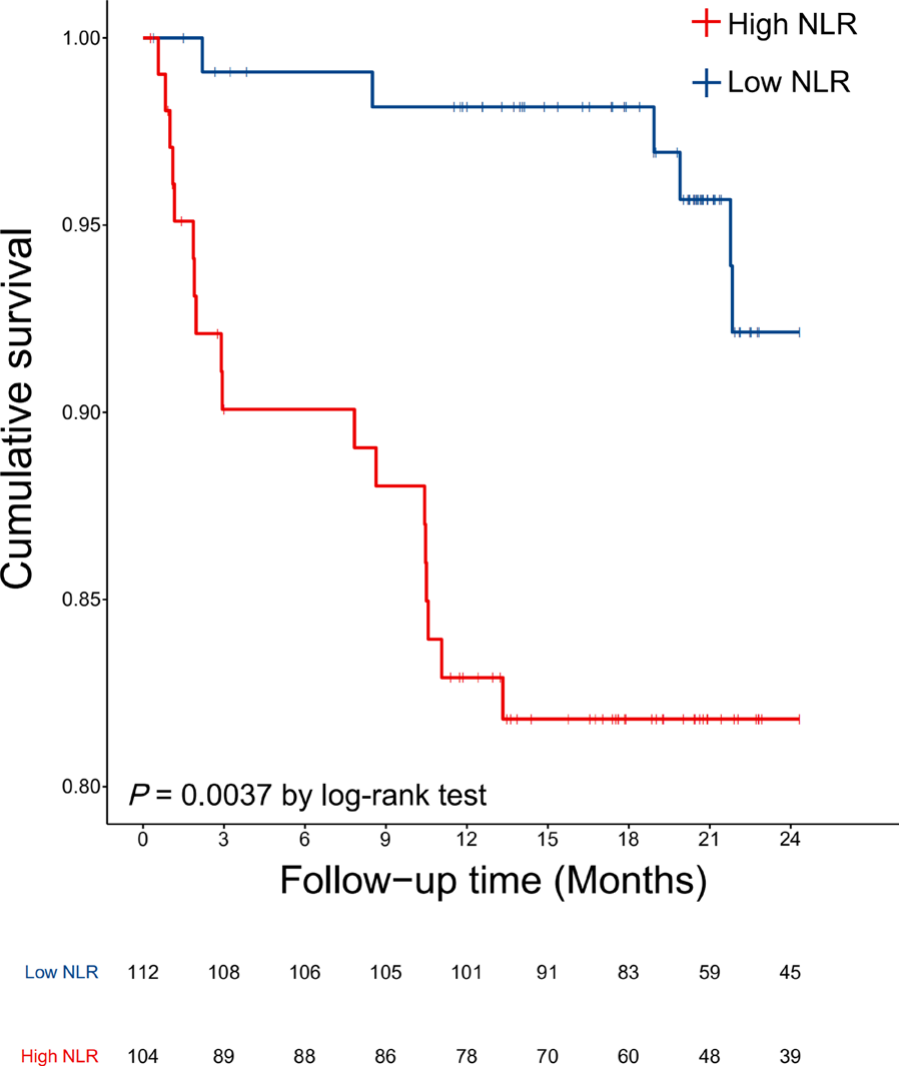
Kaplan–Meier survival analysis stratified by NLR value 4.8 (low NLR group, < 4.8; high NLR group, ≥ 4.8). The differences between the two groups was assessed with log-rank test. The freedom from early adverse events in the low NLR group was significantly higher than high NLR group (*P* = 0.004). NLR, neutrophil-to-lymphocyte ratio

Early adverse events occurred in 24 patients (11.1 %), which included one mortality due to stroke, 13 cases of type I /II endoleak, 5 cases of retrograde aortic dissection, 4 cases of aortic rupture (two died and the others survived after re-intervention) and one case of stent graft-induced new entry. The overall probability of freedom from adverse events in the high NLR group at 2 years were 82.7 % while low NLR group was 94.6 % (*P* = 0.005). (Fig. 1).

Univariate and multivariable Cox-hazard regression analyses of early adverse events in the study population were shown in Table 4. Univariate analysis indicated that platelet count preoperatively, preoperative diastolic blood pressure and NLR preoperatively were the susceptive risk factors for early adverse events (*P* < 0.2). Multivariable regression analysis showed that the NLR preoperatively (HR per SD, 1.98; 1.14–3.44; *P* = 0.015), diastolic blood pressure on admission (HR, 0.86; 95 % CI, 0.78–0.95; *P* = 0.003) had independent influences on 2-year overall event-free survival.

**Table 4:** Univariate and multivariable analysis of 2-year adverse events

Variable	Univariate analysis		Multivariate analysis	
	HR (95 % CI)	*P* value	HR (95 % CI)	*P* value
Age	1.02 (1.00, 1.07)	0.296		
Male gender	0.40 (0.13, 1.22)	0.105	0.49 (0.10, 2.31)	0.363
BMI	0.94 (0.81, 1.11)	0.479		
Smoking	0.84 (0.23, 3.06)	0.792		
Alcohol	2.82 (0.92, 8.61)	0.069	0.98 (0.23, 4.16)	0.980
Hypertension	1.60 (0.44, 5.81)	0.476		
False lumen patency				
Patent false lumen	Reference		1	
Partial thrombosis	0.67 (0.15, 3.10)	0.608	0.50 (0.07, 3.28)	0.466
Complete thrombosis	3.43 (0.74, 15.89)	0.115	4.14 (0.51, 33.35)	0.182
Distance of LSCA ≤ 2 cm	1.02 (0.28, 3.71)	0.976		
Maximum diameter of abdominal aorta (cm)	1.07 (0.97, 1.18)	0.159	1.09 (0.92, 1.29)	0.316
SBP on admission (mmHg)	1.00 (0.97, 1.03)	0.907		
DBP on admission (mmHg)	0.91 (0.83, 0.96)	0.003	0.86 (0.78, 0.95)	0.003
Temperature preoperatively (℃)	0.64 (0.14, 2.85)	0.558		
Creatinine preoperatively (µmol/l)	1.00 (0.98, 1.02)	0.741		
Platelet count preoperatively (×10^9^/l)	0.99 (0.99, 1.00)	0.107		
Preoperative NLR per SD	1.58 (1.11, 2.26)	0.011	1.98 (1.14, 3.44)	0.015
Alpha-blockers	3.60 (0.47, 7.68)	0.219		
Beta-blockers	0.95 (0.31, 2.91)	0.929		
ARBs	1.06 (0.24, 4.79)	0.939		
ACEIs	1.12 (0.23, 4.81)	0.939		
CCBs	0.47 (0.16, 1.46)	0.193	0.78 (0.14, 4.40)	0.776
Statins	0.67 (0.21, 2.16)	0.497		
Timing of procedure				
Acute (1–14 days)	Reference			
Subacute (14–92 days)	1.16 (0.39, 3.46)	0.787		
ASA classification				
I	Reference		Reference	
II	0.54 (0.17, 1.77)	0.309	0.51 (0.10, 2.73)	0.433
III	2.96 (0.57, 15.36)	0.195	3.19 (0.22, 46.42)	0.395
Adjunctive stents	3.48 (1.07, 11.32)	0.038	0.79 (0.13, 4.79)	0.800

*HR* hazard ratio,* CI* confidence interval,* BMI* body mass index,* LSCA* left subclavian artery, *NLR* neutrophil-to-lymphocyte ratio, *SBP* systolic blood pressure, *DBP* diastolic blood pressure, *ASA* American Society of Anesthesiologists

## Discussion

The aim of this study was to evaluate the relationship between preoperative NLR and early outcomes of TEVAR. For every increase of one SD in preoperative NLR value, the risk of early adverse events increased by 98 % (95 % CI, 1.14–3.44) in the multivariate adjusted model. To the best knowledge of us, this is the first research to access the predictive role of NLR in the uTBAD patients receiving TEVAR with a cut-off value 4.8.

Although long-term safety and efficacy of TEVAR have been confirmed with multiple studies [3, 5, 17], early adverse events still attract controversy and impede the extensive application of TEVAR in uTBAD. In 2019, Professor Adams and his colleagues [6] suggested that high-risk subgroup of uTBAD patients may benefit from early intervention. However, most areas in China do not have a sound medical system, which would bring difficulties to follow-up and timely risk assessment of aortic dissection. It is therefore of great value to determine the optimal timing of TEVAR procedure to evade the early adverse events.

Previous studies focused on the association between NLR value and poor clinical outcomes of type A aortic dissection (TAAD) or severe aortic events after TEVAR or open surgery [18, 19]. Lafçi et al. [20] conducted a study with 123 patients and found that a high pre-NLR was associated with in-hospital mortality with a cut-off value 8. In another study, Bedel et al.  [21] also observed an association between high levels of admission PLR and NLR and high mortality and organ dysfunction in acute TAAD patients. The studies above implied that combinations of inflammatory markers have a satisfied predictive capability for the in-hospital mortality. In contrast, in a large sample cohort study of 744 patients with TAAD, combinations of platelet, neutrophil and lymphocyte were unable to predict 30-day mortality [22]. Interestingly, when investigators combined all three biomarkers (platelet counts, lymphocyte-to-neutrophil ratio and lymphocyte monocyte ratio) in the predictive model, it provided the strongest predictive value of 30-day mortality. The reason behind this apparent contradiction might be unclear time of NLR measurement. In these studies, Azab et al. [23] used average NLR during the inpatient stay in the prediction model rather than admission, discharge, or maximum NLR. On the contrary, Park et al. [24] suggested that 24-hour NLR had a better predictive value of in-hospital mortality than the admission NLR. When compared with TAAD, TBAD showed less early postoperative mortality and complications such as renal insufficiency, liver insufficiency, and gastrointestinal hemorrhage. uTBAD Patients with strict blood pressure and heart rate control have a longer life expectancy and deserve an elective surgery instead of emergent one. King et al. [9] highlighted the importance of detection and treatment of high immune or inflammatory response in the endovascular aortic repair (EVAR) of elective abdominal aortic aneurysm (AAA). In this context, NLR 24 hours before operation is a better predictive index compared with the admission one. In our study, inflammatory index 24 hours before operation was utilized to predict the adverse events. We found significant difference of early outcomes between the high NLR group and low NLR group (adverse events, 17.3 % vs. 5.4 %, *P* = 0.005) despite that systemic inflammatory response of uTBAD is relatively lower than complicated aortic dissection or type A aortic dissection considering the lesion and comorbidities.

Although previous clinical evidence has clearly shown that the increased NLR value is associated with poor outcomes after endovascular repair, the cut-off value varies among studies [8, 10, 19, 25, 26]. Octeau et al. [27] defined the median NLR 3.5 for all patients as the cut-off value in a retrospective study including 107 patients undergoing thoracic endovascular aneurysm repair. In another study, King et al. [9] used a ROC curve analysis to determine the cut-off value of NLR 4.0 with the highest specificity and sensitivity to correlate with mortality. In the present study, cut-off value 4.8 was obtained using a time-dependent ROC curve associated with 2-year poor outcomes. As NLR is a continuous dynamic value rather than a dichotomous variable, King et al. [9] used a tertile analysis, in which highest NLR group showed poorest outcomes compared with the lower two groups. In our study, effect of NLR per SD change was evaluated in the univariate and multivariable analysis (Table 4). It showed that the risk of early adverse outcomes increased by 98 % for every increase of one SD in preoperative NLR (multivariate HR, 1.98; 1.14–3.44; *P* = 0.015). Although there is a need to perform experiments with more subjects, this study shows potential risk of early adverse events for uTBAD patients undergoing TEVAR with a higher NLR than 4.8.

The effect of inflammatory markers in cardiovascular diseases has been studied routinely and a credible relationship between inflammatory markers and cardiovascular diseases has been confirmed [8, 28, 29]. Although the exact mechanisms of these markers are not well understood, it is thought that they reflect the complex interaction between the local immune response at the microenvironment of aortic dissection and the systemic inflammatory response [29–31]. The etiology of AD is complex, in which inflammation plays an important role [32, 33]. After onset, the systemic response to injury causes neutrophilia and massive neutrophil accumulation in the tunica adventitia of the dissected aorta [34]. The activation of neutrophils and endothelial cell adhesion to generate a large amount of reactive oxygen intermediate, in turn, increasing the vascular endothelial damage, and the excessive consumption of platelets after thrombosis may also increase the risk of aortic dissection rupture [35]. Clinical evidence has confirmed that systemic inflammatory markers like NLR is independently associated with mortality of AD patients [20, 25, 36, 37]. Taken together, inflammatory markers show promise in the diagnosis, timing of treatment and prognosis of AD. However, inflammatory markers are not factored into any risk stratification model of AAD until now [38]. It would be of interest to take the NLR value into consideration of predictive model of AAD for secondary prevention.

## Limitations

Firstly, due to the retrospective study cohort from single center and small number, results may be biased. Secondly, details of adverse events could not be demonstrated via telephone. Thirdly, in clinical practice, however, there remains some controversy regarding the endovascular repair of uTBAD. Although routine TEVAR in uTBAD is standard practice at our institution, different TEVAR procedure manipulations and medical treatment would also affect the results. Fourthly, uTBAD patients who refused to endovascular repair and received optimal medical treatment alone were not included in the study, which may overstate the efficacy of endovascular repair of uncomplicated TBAD. Lastly, anatomic characteristics like oversizing were not included in the analysis, which demand a multi-center study in the future.

## Conclusions

Current study revealed that high preoperative NLR value is an independent risk factor of early adverse events in uTBAD patients undergoing TEVAR. Further studies focused on predictive model combined with NLR value should be conducted to open new perspectives in the field of secondary prevention.

## Data Availability

The datasets used and/or analysed during the current study available from the corresponding author on reasonable request.

## Abbreviations

TEVAR: Thoracic aortic endovascular repair
uTBAD: Uncomplicated type B aortic dissection
IQR: Interquartile range
NLR: Neutrophil-to-lymphocyte ratio
LSCA: Left subclavian artery
COPD: Chronic obstructive pulmonary disease
CAD: Coronary artery disease
TAAD: Type A aortic dissection
EVAR: Endovascular aortic repair
AAA: Abdominal aortic aneurysm
PIS: Post-implantation syndrome
